# Cholera Toxin B Subunits Assemble into Pentamers - Proposition of a Fly-Casting Mechanism

**DOI:** 10.1371/journal.pone.0015347

**Published:** 2010-12-21

**Authors:** Jihad Zrimi, Alicia Ng Ling, Ernawati Giri-Rachman Arifin, Giovanni Feverati, Claire Lesieur

**Affiliations:** 1 LAPTH, Université de Savoie, CNRS, Annecy le Vieux, France; 2 National University of Singapore, Physics Department, Singapore, Singapore; 3 Institut Teknologi Bandung (ITB), School of Life Sciences and Technology, Bandung, Indonesia; 4 CEA-CNRS-Université Joseph Fourier, IRTSV, LBBSI, Grenoble, France; Griffith University, Australia

## Abstract

The cholera toxin B pentamer (CtxB_5_), which belongs to the AB_5_ toxin family, is used as a model study for protein assembly. The effect of the pH on the reassembly of the toxin was investigated using immunochemical, electrophoretic and spectroscopic methods. Three pH-dependent steps were identified during the toxin reassembly: (i) acquisition of a fully assembly-competent fold by the CtxB monomer, (ii) association of CtxB monomer into oligomers, (iii) acquisition of the native fold by the CtxB pentamer. The results show that CtxB_5_ and the related heat labile enterotoxin LTB_5_ have distinct mechanisms of assembly despite sharing high sequence identity (84%) and almost identical atomic structures. The difference can be pinpointed to four histidines which are spread along the protein sequence and may act together. Thus, most of the toxin B amino acids appear negligible for the assembly, raising the possibility that assembly is driven by a small network of amino acids instead of involving all of them.

## Introduction

The function of a vast majority of proteins depends on their capacity to self-assemble, either transiently or permanently, into oligomers [1], [2]. Understanding the mechanism of protein assembly is particularly important due to its implication in numerous pathologies from bacterial infection (e.g. Bacillus Anthracis) to protein misfolding diseases (e.g. Alzheimer, Parkinson) [3], [4].

The AB_5_ toxin family is a good example of the role of protein assembly in providing molecules their capacity to kill [5], [6]. The AB_5_ toxins are hetero-hexameric assemblies comprising a toxic single A-subunit and a “carrier” pentamer of B-subunits (Cholera toxin, Ctx; Heat-labile enterotoxin, LT; shiga-toxin, shiga-like toxins, also called verotoxin and Pertussis toxin, PT). The x-ray structures of all the AB_5_ members are available and share a remarkable degree of structural homology despite the lack of sequence homology of their A and B subunits, respectively [7], [8], [9], [10]. The B subunits can be produced by the bacteria, in the absence or in the presence of the A subunit, into pentamers that are structurally and functionally indistinguishable. This implies that the sequence of the B subunit contains all the necessary information for its assembly. These findings have supported the use of *in vitro* approaches to investigate the assembly of the B subunit alone.

Among AB_5_ members, the *in vitro* disassemblies of the cholera toxin B subunit (CtxB_5_) and of the human heat-labile enterotoxin B subunit (LTB_5_) are well documented [11], [12], [13], [14], [15], [16], [17]. The two toxins *in vitro* assemblies have also been reported [18], [19], [20], [21], [22]. Despite high sequence identity (84%), similar 3D structures and similar functions, the two toxins present different disassembly and reassembly features, even when performed under identical experimental conditions.

The two toxins have different stabilities and pH-sensitivities, with the threshold for pH disassembly being 1.9±0.06 and 3.7±0.09 for LTB_5_ and CtxB_5_, respectively [15], [16].

The reassembly of CtxB_5_ is concentration-dependent up to 26 µM whereas the reassembly of LTB_5_ is already concentration-independent above 6 µM [18], [19]. Thus, at low concentration, the association steps are rate-limiting only in the reassembly of CtxB_5_. We previously proposed a model of the CtxB_5_ reassembly, which explained the slow association rate by taking into account the isomerization of the Pro 93. The pro 93 is in a *cis* conformation in the native state of CtxB_5_.

However the difference in the association rates between the two toxins cannot be solely explained by the isomerization of Pro 93, which is also in a *cis* conformation in LTB_5_, unless the two toxins have two different proline isomerization rates. The rate of proline isomerization is sensitiveto the side chain of the amino acid located upstream the proline [23]. There is a threonine residue at position 92 in both the toxins so the isomerization rate of the Pro 93 is likely to be similar for the two toxins.

The goal of the present work was to rationalize the two different association rates observed for the assembly of the two toxins. One possibility was that a reaction, unique to the reassembly of CtxB_5_, was slowing down the subunit association so that the effect of the Pro 93 isomerization on the association rate becomes apparent. In absence of such a reaction in the reassembly of LTB_5,_ the effect of the proline isomerization rate on the association of LTB subunits would not be detectable.

To test this hypothesis, the detailed study of CtxB assembly was undertaken. The CtxB_5_ reassembly was inhibited over the pH range 5.0 to 8.0, with a pKa around 6.0. The protonation at low pH prevented three different steps: (i) the acquisition of a 3D intramolecular rearrangement required for the toxin monomer to associate (conformational gating), the formation of one of the two interfaces present per subunit and finally a transition from a non native to a native pentameric conformation. Experimental and simulation based data indicated the involvement of the protonation of histidine residues in the association pH-dependent steps.

## Methods

### Reagents and buffers

Cholera toxin B pentamer (CtxB_5_), anti CtxB_5_ polyclonal antibody, HRP-goat anti-rabbit secondary antibody and all other chemicals were obtained from Sigma. McIlvaine buffer (0.2 M disodium hydrogen phosphate, 0.1 M citric acid, pH 5.0–8.0), PBS and 0.1 M KCl/HCl at pH 1.0 were used. All buffers were filtered through sterile 0.22 µm filter before use.

### SDS-PAGE analysis

SDS-PAGE (15% or 12%) was performed with a Bio-Rad mini-Protean 3 system using the Laemmli method [24]. The gels were Coomassie blue or silver stained. 1 µg of sample was loaded on each lane of the gel.

### Disassembly of CtxB_5_

The conditions used for disassembly were adapted from elsewhere [19]. Briefly, lyophilized native CtxB_5_ was dissolved in PBS at a concentration of 344 µM and was diluted in 0.1 M HCl/KCl at pH 1.0 at a final concentration of 8.6 µM, for 15 min.

### Reassembly of CtxB_5_

The conditions used for reassembly were adapted from elsewhere [19]. Briefly, native CxtB_5_ was acidified in 0.1 M HCl/KCl at pH 1.0 for 15 min at a final toxin concentration of 86 µM. The toxin was subsequently diluted to specified final concentration, in McIlVaine buffers at pH ranging from 5.0 to 8.0 to promote reassembly. The samples were incubated for a maximum of 60 min at 23°C. The pH of the samples was measured with a microelectrode. Immediately after the addition of the McIlVaine buffer and after incubation for specific times, samples were removed from the reaction mixture for analysis. The presence of CtxB intermediates (Dimer, trimer, tetramer and pentamer) were detected by GM1-Elisa and by cross-linking experiments combined with SDS-PAGE [19]. The kinetics of their formation was measured by Trp-fluorescence [19]. The 3D conformation of the samples was also monitored by fluorescence spectroscopy. The native quaternary structure of the toxin was inferred from SDS-PAGE analyses since CtxB_5_ is stable in SDS-containing buffers and migrates in the gel with an apparent molecular weight characteristic of the B-subunit pentamer. This indirectly provided some information on the stability of the toxin pentamer. Only the native pentamer is SDS-resistant. The CtxB concentration for all experiments refers to the monomeric concentration.

### Trp-Fluorescence

Fluorescence measurements were performed using a Cary eclipse Varian spectrofluorimeter. Measurements were made at a toxin concentration of 8.6 µM or of 35 µM. Samples of native CtxB_5_ at 344 µM concentration were acidified in 0.1 M HCl/KCl buffer at pH 1.0 for 15 min and added directly in the cuvette containing McIlVaine buffers at a pH range 8.0–5.0. Measurements started immediately after the addition of the samples in the cuvette. Excitation was at 295 nm, with emission recorded at 349 nm and slit widths of 2.5 and 10 nm for excitation and emission, respectively. When emission spectra were recorded, the Raman contribution for water was removed by subtraction of a buffer blank. The time delay between the injection and the collection of the first data points was less than 10 seconds. The experiments were carried out at 23°C.

### Far-UV Circular Dichroism

Far-UV circular dichroism spectra were recorded on a Jasco J810 spectropolarimeter. A cell with a path length of 0.2 cm, a spectral band width of 4 nm, and a time constant of 1 s were used, and each spectrum was recorded as an average of 5 scans. Native CtxB_5_ samples at 344 µM concentration were acidified in 0.1 M HCl/KCl buffer at pH 1.0 for 15 min (4× dilution) and added directly to the cuvette containing McIlVaine buffers at pH 7.0 or 5.0 or containing 0.1 M HCl/KCl buffer at pH 1.0. The native CtxB_5_ was diluted 4× into PBS prior addition to the cuvette containing McIlVaine buffer at pH 7.0 or pH 5.0. The final protein concentration in the cuvette was 8.6 µM. The scans were measured immediately with a time delay between the injection and the data collection of less than 10 seconds. The experiments were performed at 23°C. Below 210 nm the data were too noisy to be collected.

### GM1-Elisa

GM1 is the cellular receptor of both LTB_5_ and CtxB_5_ [25]. In a reassembly mixture, the amount of CtxB species that have acquired the ability to bind to GM1 was determined using GM1-Elisa as previously described [18], [19]. CtxB dimer, trimer tetramer and non native pentamer as well as native pentamer are recognized and captured by GM1 [19].

### Chemical cross-linking and SDS-PAGE analysis of the oligomeric state of CtxB_5_

The cross-linking conditions were adapted from elsewhere [19]. The CtxB samples, after acidification or subsequent dilution in McIlVaine buffers, were mixed with 0.1 M Tris at pH 8.0 and immediately cross-linked with a final concentration of 4% glutaraldehyde for 2 min at 23°C (v/v). Thus, the samples were cross-linked at similar pH. After cross-linking, the native CtxB_5_ ran as 5 bands corresponding to the apparent molecular weights, in decreasing order, of the cross-linked CtxB pentamer, tetramer, trimer, dimer and monomer. The CtxB cross-linked intermediates issued from the native CtxB_5_ sample, appeared only if the sample was boiled. They corresponded to partially cross-linked pentamer which are dissociated upon boiling [19].

### SDA (Simulation of Diffusional association)

This software simulates the Brownian dynamics of two proteins in water and is used to test the kinetics of the formation of a protein interface [26]. Association rates are computed by counting the number of hits (encounters) per second in many BD (Brownian dynamics) trajectories. The simulation of a trajectory started with the two proteins (two adjacent chains of CtxB_5_ are considered) at uniformly random orientations at a center-to-center separation distance b, and finished when the protein centers reached a separation distance c greater than b (b = 100 Å, c = 500 Å). At distance b and greater there is no interaction between proteins because of the solvent polarity. 20 000 BD trajectories were simulated to achieve a statistical error of 10% in the computed association rates.

The hit of the two proteins (encounter) is defined by monitoring the formation of a given subset of the atomic contacts observed in the x-ray structure of the protein complex (CtxB_5_). Native contacts are defined by firstly, tabulating all intermolecular donor-acceptor atom contacts shorter than a center-to-center distance dmax (4.5 Å) in the x-ray structure of the protein complex and secondly, by generating a table listing dependent pairs of contacts, i.e. those contacts in which either atom is within dmin (<6 Å) of an atom of another contact within the same protein. During a BD trajectory, the donor-acceptor atomic contacts shorter than dc are monitored (dc = 50 Å). Then the number of independent contacts (those contacts in which either atom is outside dmax (>6 Å) of an atom of another contact within the same protein) is computed by ensuring that no more than one contact from any dependent pair of contacts is counted. This was the reaction criterion (Table 1) used for computing the association rates (see details within [26]. The detailed equations for calculating the rate of association are well-described in [26].

**Table 1:** Structural parameters for the CtxB_5_ and LTB_5_ complexes.

PDB	Resolution (Å)	Reaction pairs^a^	D_tr_ (A^2^/ps)^b^	D_rot1_ (rad^2^/ps)^c^	D_rot2_ (rad^2^/ps)^d^
1EEI	2	40 (20)	0,027	3.92 10^−5^	3.92 10^−5^
1LTR	3.04	47(18)	0,027	3.92 10^−5^	3.92 10^−5^

a Number of intermolecular donor-acceptor pairs within 5 Å in the crystal structure of the complex. The number of independent donor-acceptor pairs is given in parentheses.

b Relative translational diffusion coefficient.

c Rotational diffusion coefficient of protein 1.

d Rotational diffusion coefficient of protein 2.

In this simulation protocol, both proteins are treated at atomic-detail interacting in a implicit solvent (water) via excluded volume. The pH of the solvent is included by changing the protonation level of surface amino acids; after, electrostatic interactions are computed by numerically solving the Poisson-Boltzmann equation (APBS software) (see details within [26].

Relative translational and rotational diffusional constants are listed in Table 1. They were assigned using the Stokes-Einstein relationships assuming a solvent temperature of 25°C and using calculated effective radii for the proteins. These radii are defined as the distance from the center to a surface where the atomic density is two thirds the density at the center of the protein.

### Protein Structures

The crystallographic coordinates of the CtxB_5_ and of the LTB_5_ complexes was extracted from the Brookhaven data bank [27] (PDB codes: 1EEI and 1LTR). Two adjacent chains are considered in the simulation. Mutants were modeled by replacing the side-chain of the mutated residue using the PDBviewer program [28].

## Results

The two toxin B pentamers different association rates are most likely due to amino acids which are different in the two toxin sequences. The most noticeable sequence differences are on charged residues. The residues 18, 94 and 102 are a tyrosine, an asparagine and a glutamic acid for LTB_5_ but two histidines and an alanine for CtxB_5_, respectively. In addition D7 and E83 are replaced by E7 and D83 in LTB_5_. Apart from one polar residue replaced by an hydrophobic residue at position 1 and vice versa at position 10, the other differences (10 more) between CtxB_5_ and LTB_5_ sequences, respectively, are keeping identical chemical properties [6].

The position of the two additional histidines in CtxB_5_ are particularly interesting as both residues, His 18 and His 94, are located upstream the β-strands constituting the protein interfaces. They could therefore have some influence on the formation of the interfaces, consequently affecting the association rate.

To investigate the role of charged amino acids, the effect of pH on the toxin reassembly was tested *in vitro* using well-established immunochemical, electrophoretic and spectroscopic methods [18], [19].

### Effect of the low pH on the reassembly of CtxB_5_

**Figure 1:**
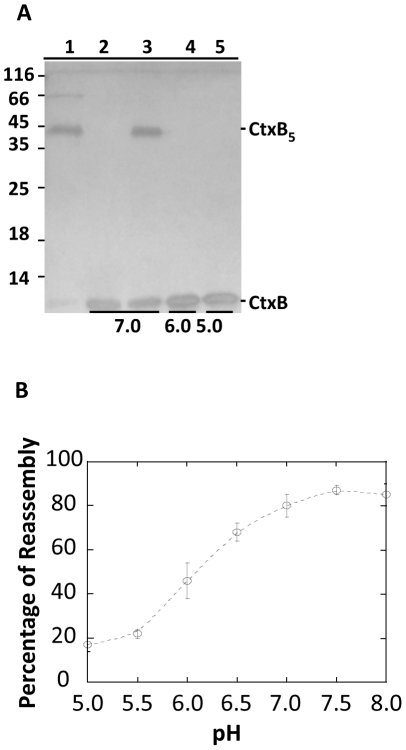
Reassembly of CtxB as function of pH. **A. Reassembly of CtxB into SDS stable pentamer.** Equal amount of reassembled CtxB was applied on each lane of a SDS-PAGE. CtxB reassembled for 0 min and 30 min at pH 7.0 (lanes 2 and 3), or for 30 min at pH 6.0 (lane 4) or and at pH 5.0 (lane 5). Lane 1 is the native CtxB_5_. Molecular weight standards are indicated in kDa on the left of the gel. The respective apparent positions of the native CtxB pentamer and of the CtxB monomer are indicated on the right of the gel. **B. Reassembly of CtxB into species capable of recognizing GM1.** CtxB_5_ (○) was treated at pH 1.0 for 15 min and subsequently diluted to a final concentration of 8.6 µM in McIlVaine buffers at indicated pH. The samples were incubated for 30 min at 23°C and analyzed by GM1-Elisa. The results of three independent experiments are shown as a mean ± S.D.

The pH dependence of CtxB_5_ reassembly was studied, after a pre-treatment of 15 min at pH 1.0, and subsequent incubation at a final toxin concentration of 8.6 µM for 30 min in McIlVaine buffers at pH ranging from 5.0 to 8.0. The reassembly of CtxB into SDS stable pentamer was monitored by SDS-PAGE (Fig. 1A). Just after dilution in McIlVaine buffer at pH 7.0 (lane 2), no SDS-stable CtxB pentamers was observed on the gel, but 30 min later (lane 3), they appeared, showing that reassembly had taken place. In contrast, when the toxin was diluted in McIlVaine buffers at pH 6.0 and at pH 5.0, no SDS-resistant pentamer was observed on the gel, even after 30 min incubation (lanes 4 and 5, respectively). So, the reassembly of CtxB into SDS-resistant pentamer was inhibited at pH below 7.0.

The reassembly of CtxB in species capable of binding to GM1 was studied by GM1-Elisa. The results showed that below pH 5.0, negligible reassembly was observed (data not shown). Between pH 5.0 and 8.0, the amount of reassembled CtxB capable of binding to GM1 increased with increasing pH (not shown). The yield of the reassembly of CtxB was plotted against pH (Fig. 1B). The yields of CtxB reassembly increased with increasing pH and described a sigmoid curve with an inflexion point around pH 6.0.

Such a mid-point pH was consistent with the deprotonation of histidine residue(s) (pKa∼6.1) and/or of the N-terminal of the toxin (pKa∼7.0).

A N-terminal DEPC-modified toxin had similar pH-dependence than the non modified toxin (unpublished results). Since DEPC attachment prevents the protonation of the modified residue, the protonation of the N-terminal at low pH appears unlikely, thus supporting the role of histidine residues in the assembly mechanism.

### Steps inhibited by the low pH

**Figure 2:**
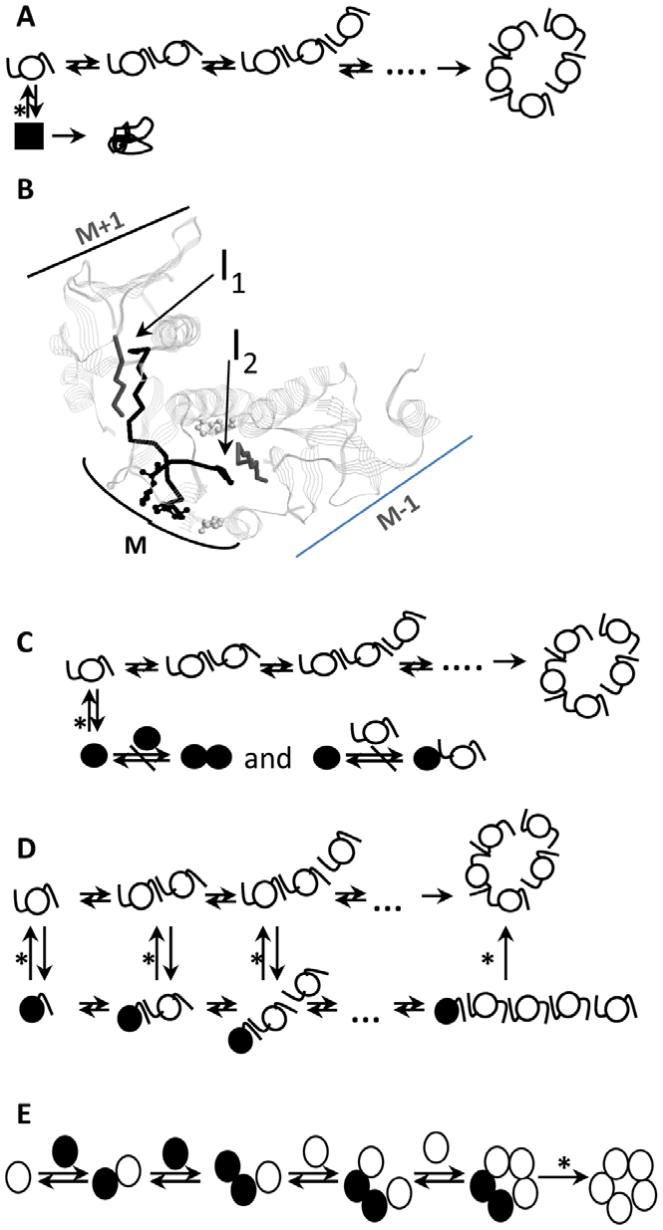
Scenari of the steps possibly inhibited by the low pH during the reassembly of CxtB. Each monomer is represented by a circle. The deprotonated and the protonated CtxB monomers are indicated in white and in black, respectively. In scenari 1, 2 and 3, the β-strands constituting the two subunit interfaces (25–33 a.a. and 97–103 a.a.) are indicated by a line only when they are capable of associating. If the association is impaired by the low pH, the strands of the interfaces are not represented. The native CtxB_5_ is represented as a ring of five monomers according to the x-ray crystallographic structure (*10*). **2A. Scenario 1.** The folding of the CtxB monomer is inhibited by the low pH. The protonated (black square) and the deprotonated CtxB (white circle) monomers have two different folds, and only the deprotonated CtxB monomer persue the assembly process. The protonated CtxB monomer misfolds irreversibly. **2B. CtxB_5_ interfaces and histidine residues.** For simplicity, out of the five CtxB monomers that composed the native pentamer, only three are shown in strands. Each monomer has two interfaces (Interfaces 1 and 2) involving two different β-strands. The strand number 3 of M (residues 25 to 33) associates with the C-terminal end of the β-strand number 6 of monomer M+1 (residues 97 to 103) to form the interface 1 (I_1_). The C-terminal end of the β-strand number 6 of monomer M associates with the strand number 3 of monomer M-1 to form the interface 2 (I_2_). The four histidine residues are indicated as balls and sticks, histidines 18 and 94 which are located upstream the two β-strands of the interfaces are colored in black. The figure was made using rasmol and using the coordinates from the x-ray structure of CtxB_5_ (*10*). **2C. Scenario 2.** The formation of both the interfaces 1 and 2 is inhibited by the low pH. The protonated CtxB monomer is association-incompetent. Only the deprotonated CtxB monomer can associate. **2D. Scenario 3.** The formation of either interface 1or 2 is inhibited by the low pH. The CtxB protonated can form only one of the two interfaces and is association-deficient. **2E. Scenario 4.** An intramolecular rearrangement (folding) within the CtxB pentamer is inhibited by the low pH. Both the protonated and the deprotonated CtxB monomers can associate together (black and white) or separately (white-white or black-black). The formation of the native pentamer is pH-dependent. In each scenario, the steps which involve deprotonation/protonation of the CtxB subunit are indicated by a star (*). The native pentamer is considered the most stable species of the reaction and therefore its formation is assumed irreversible.

The steps that could be inhibited by the low pH are illustrated on schematics in figure 2. The first possibility is the inhibition of the proper folding of the CtxB monomers (Fig. 2A, scenario 1). If protonated CtxB monomers misfold, the amount of deprotonated CtxB monomer which remains available decreases at low pH, leading to a reduction in the yield of the toxin reassembly. The second possibility is the inhibition of the association between CtxB subunits. Each subunit associates with two others through the formation of two different interfaces (Fig. 2B). Interface 1 (I_1_) involves the association between the β-strand number 3 of subunit M, composed of the residues 25 to 33 and the C-terminal end of the β-strand number 6 of subunit M+1, composed of the residues 97 to 103. Interface 2 (I_2_) involves the association between the C-terminal end of the β-strand number 6 of subunit M and the β-strand number 3 of subunit M-1. Therefore the low pH can inhibit the association of one subunit with the two others or with only one other. In the former case, the protonation prevents the two β-strands from associating and consequently only deprotonated CtxB monomer are able to associate (Fig. 2C, scenario 2). As in the first scenario, this would lead to a reduction of the CtxB monomer capable of associating (deprotonated CtxB monomer) at low pH and to a lower yield of reassembly. In that scenario, protonated CtxB monomers are association-incompetent. In the case that the protonation prevents the formation of only one of two interfaces (Fig. 2D, scenario 3), both deprotonated and protonated CtxB monomers are able to associate. Deprotonated CtxB monomer can associate with both protonated and deprotonated CtxB monomer whereas protonated CtxB monomer can only associate with deprotonated CtxB monomer. The protonated CtxB monomer is in that case only association-deficient. Both scenari 2 and 3 are conceivable particularly since there is one histidine residue located upstream each one of the β~strands of the interfaces.

The fourth possibility is the inhibition of the formation of native pentamers (Fig. 2E, scenario 4). In that case, both protonated and deprotonated CtxB monomers associate and form pentamers. But the pentamer composed of one or several protonated CtxB monomers has a non native pentameric conformation. The transition to a native pentamer would require deprotonation of the residue(s).

The existence of two populations of CtxB monomers, one protonated and one deprotonated, is implicite in all scenari.

### Proper refolding of the CtxB monomer is inhibited at low pH

**Figure 3:**
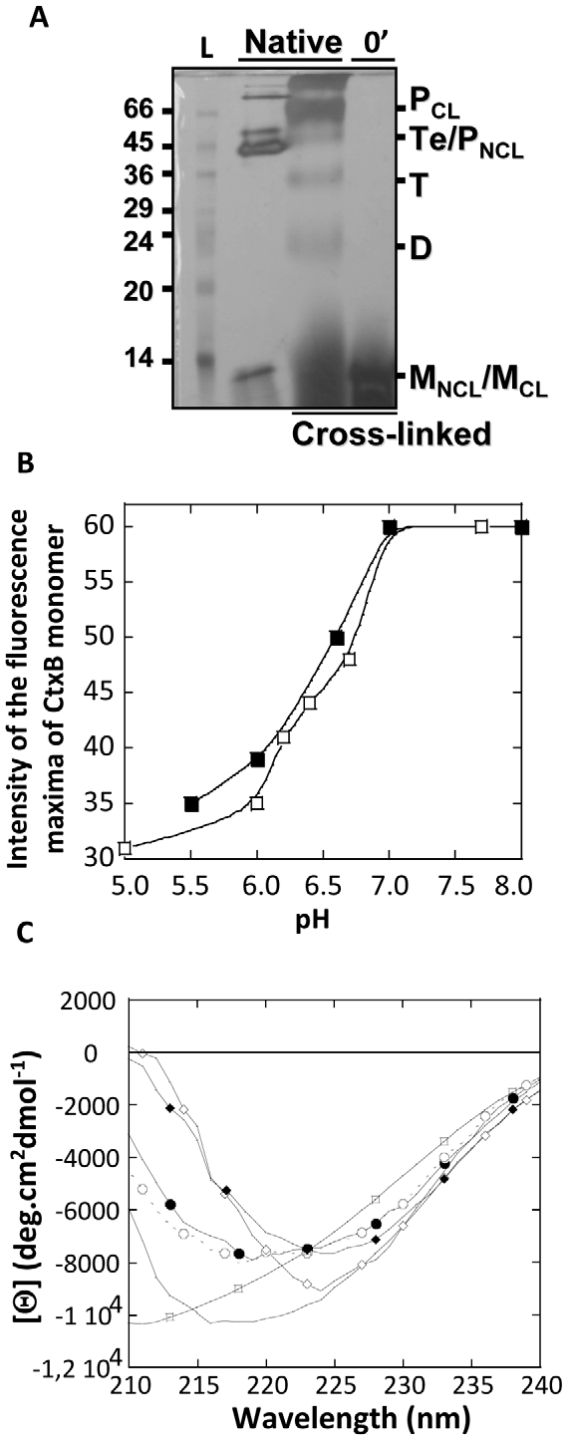
Conformational changes in the structure of the CtxB monomer as function of pH. CtxB_5_ was treated for 15 min at pH 1.0 and subsequently diluted to a final concentration of 8.6 µM, or otherwise specified, in McIlVaine buffers at indicated pH **A. CtxB monomeric state.** After acidification and just after dilution in McIlVaine buffer at pH 7.0, CtxB was cross-linked (0′) with glutaraldehyde for 2 min prior mixing with 4× sample buffer and subsequent analysis by SDS-PAGE. The same treatment was performed on native CtxB_5_, to indicate the position of cross-linked CtxB monomer (M_CL_), dimer (D), trimer (T) tetramer (Te) and pentamer (P_CL_) (*23*). Native, not cross-linked CtxB_5_, was also loaded on the gel to indicate the position of the native pentamer without cross-linking (P_NCL_). Some of the native CtxB_5_, not cross-linked, did not resist the SDS treatment after boiling and dissociated into monomer, indicating the apparent molecular weight of the non cross-linked monomer (M_NCL_). L stands for the apparent molecular weights of standards, indicated in kDa. Only the cross-linked samples were boiled prior loading. **B.** pH dependences of the fluorescence intensity of CtxB monomers just after dilution in McIlVaine buffer at indicated pH, for reassemblies at 8.6 µM (□) and at 35 µM (▪). a.u. stands for arbitrary unit. The data for the toxin concentration at 35 µM were divided by four to allow comparison with the data for the toxin concentration at 8.6 µM on the same graph. **C. Far-UV CD spectra.** Native CtxB_5_ at pH 7.0 (•) and at pH 5.0 (♦). Native CtxB_5_ was acidified for 15 min at pH 1.0 and diluted into McIlVaine buffers at pH 7.0, just after dilution (—) (CtxB monomer), or 30 min later (○) (reassembled CtxB) or just after dilution at pH 5.0 (◊). (□) acidified CtxB at pH 1.0 for 15 min. The toxin concentration is 8.6 µM.

Hence, we first looked for evidences of the presence of two CtxB monomeric populations. After treatment at pH 1.0 for 15 min and immediately after subsequent dilution in McIlVaine buffers at pH 7.0, there was no reassembly of the CtxB monomer into dimer or intermediates of higher stoichiometry (Fig. 3A, lane 4).

Thus immediately after neutralization, CtxB was essentially monomeric. Since the highest yield of reasssembly was at pH 7.0, it was reasonable to assume that immediately after dilution at pH lower than 7.0, CtxB was also mainly monomeric. It was therefore possible to study the conformation of the sole CtxB monomer, after the toxin acidification and immediately after its dilution in McIlVaine buffers at pH 7.0 and lower.

The effect of the pH on the conformation of the CtxB monomer was first analyzed using Trp fluorescence. Trp-88 is the unique tryptophan presents in CtxB_5_. CtxB_5_ was treated at pH 1.0 for 15 min and subsequently diluted in McIlVaine buffers at pH ranging from 5.0 to 8.0, to final toxin concentrations of 8.6 µM or of 35 µM. The Trp-fluorescence scans were measured immediately after addition of the acidified CtxB sample into the cuvette containing the McIlVaine buffer (not shown). The intensity of the Trp-fluorescence maxima of the CtxB monomer was plotted against pH (Fig. 3B). On the plot of Fig. 3B, the intensities of the Trp-fluorescence maxima of the toxin sample at 35 µM have been divided by four for the sake of comparison with the data obtained at 8.6 µM. The Trp-88 signal increased with increasing pH in a sigmoid fashion similarly at both toxin concentrations and with a similar inflection point at around 6.5. This event was concentration-independent and therefore due to an intramolecular reaction. It was reminiscent of the quenching of the Trp 88 fluorescence observed on CtxB pentamer at pH 5.0 and on folded CtxB monomer reported previously by De Wolf [12]. The quenching was attributed to the protonation of histidine residues, more likely His 94 and/or His 13 which are in close proximity to Trp 88. There was no Trp-fluorescence quenching on unfolded CtxB monomer. In order to assess if the Trp-88 fluorescence increases with the pH was similarly due to the deprotonation of histidine residues, the conformational state of the CtxB monomer was investigated using far-UV circular dichroim measurements.

At pH 7.0, the Trp-88 fluorescence of the CtxB monomer was significantly reduced compare to that of the native pentamer at pH 7.0 (300 arbitrary unit) due to the dissociation of the CtxB as previously shown [19].

The far-UV spectra of CtxB_5_ and of CtxB monomers were measured at pH 7.0 and at pH 5.0 (Fig. 3C). The CtxB monomers were prepared as for the Trp-fluorescence experiment. It was not possible to estimate the secondary structure contents of the samples because of the low reliability of the data measured below 210 [29]. A typical far-UV spectrum of mixed α and β–structures was obtained for the native CtxB_5_ at pH 7.0 [29]. Now, the overall aspect of the far-UV spectra of the CtxB monomers at both the pH and of the CtxB pentamer at pH 5.0, markedly differed from that of the native CtxB_5_ at pH 7.0 and from each other. The ellipticity at 222 nm, characteristic of the peptide bond and of changes within the secondary structures, was similar for the native pentamer at pH 7.0 (Θ_222 nm_ = −7525 deg.cm^2^.dmol^−1^) and for the pentamer at pH 5.0 (Θ_222 nm_ = −7318 deg.cm^2^.dmol^−1^), indicating no major changes in the secondary structure of the pentamer at pH 5.0. In contrast, the ellipticity at 222 nm of the CtxB monomers at pH 7.0 (Θ_222 nm_ = −9856 deg.cm^2^.dmol^−1^), and at pH 5.0, (Θ_222 nm_ = −8550 deg.cm^2^.dmol^−1^), underwent detectable changes indicating that the two toxin monomers had different and non-native secondary structures. However, both of the two CtxB monomers adopted a partially folded state and not a fully unfolded state as none had a far-UV spectrum typical of a fully unfolded state [29].

At pH 5.0, the far-UV spectra of the pentamer and of the monomer showed a significant shift of the wavelength maximum (peak centered to 224 nm) compared to the native one (peak centered to 219 nm). This peculiar CD signal could be due to the contribution to the far-UV CD of conformational changes in or in the environment of the side chain of a disulfide bridge, as previously reported for lysosome [30]. The disulfide chromophore can strongly affect the sign, the amplitude and the wavelength of the maximum of the far-UV CD signal when its dihedral angle deviates from the 90° position in a close packed environment [30]. In contrast, in absence of structure around the disulfide bridge (unfolded state), its side chain participation to the far-UV signal becomes negligible [30]. There is a unique disulfide bridge in CtxB_5_ that links Cys 9 to Cys 86. Thus, at pH 5.0, in the CtxB pentamer and in the protonated CtxB monomer, there might be some structural organization that maintains the local packing around that disulfide bridge giving rise to the strong negative peak centered to 224 nm. This was supported by the absence of such a peak after acidification of CtxB_5_ at pH 1.0 for 15 min (Fig. 3C). The CtxB monomer acidified at pH 1.0 had a far-UV CD spectrum indicating some 2D reorganization (Θ_222 nm_ = −7886 deg.cm^2^.dmol^−1^) but yet not the loss of the 2D structures associated with a fully unfolded state.

Maintenance of some structural organization within the vicinity of the disulfide brigde in the CtxB monomer, was consistent with the quenching of Trp-88 by the protonated His 13 and/or His 94 as His 13, His 94 and Trp-88 are within a 13 Å radius of the Cys-9-Cys-86 disulfide bridge, in the native CtxB_5._

After 30 min at pH 7.0, the far-UV CD spectrum was almost identical to that of the native CtxB_5_ at pH 7.0, indicating significant recovery of the native secondary structure upon reassembly (Fig. 3C).

So the protonated CtxB monomer at pH 5.0 and the deprotonated CtxB monomer at pH 7.0 had two different non-native conformations. We then checked if the protonated CtxB monomer was capable of associating.

### Inhibition of the formation of CtxB oligmeric intermediates at low pH

The capacity of the CtxB monomer to associate was tested by measuring the formation of CtxB oligomeric intermediates (dimer, trimer, tetramer) by cross-linking combined to SDS-PAGE and by measuring the time-dependent changes in the intensity of the Trp-fluorescence maxima, which takes place during the CtxB reassembly [19].

**Figure 4:**
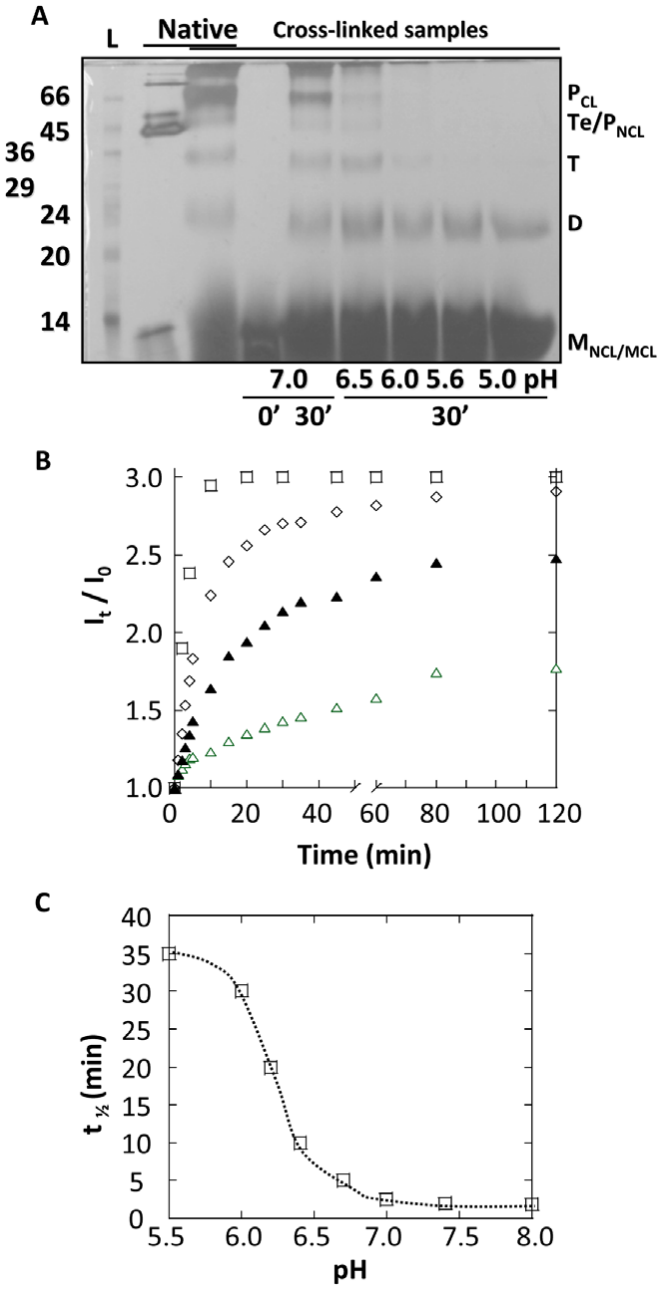
Formation of CtxB intermediates as function of pH. CtxB_5_ was treated for 15 min at pH 1.0 and subsequently diluted to a final concentration of 8.6 µM in McIlVaine buffers at indicated pH. The samples were incubated for 30 min at 23°C. **A.** **Determination of the CtxB intermediates by cross linking and SDS PAGE.** Immediateley after neutralization or 30 min later, the samples were cross-linked with glutaraldehyde for 2 min prior mixing with 4× sample buffer and subsequent analysis by SDS-PAGE. Cross-linked CtxB Pentamer (P_CL_) and stoechiometric intermediates (D, dimer; T, trimer; Te, tetramer) as well as cross-linked CtxB monomer (M_CL_) were identified on the gel. Native CtxB_5_ cross-linked (P_CL_) and not cross-linked (P_NCL_) were also loaded on the gel to identify their respective position. L stands for the apparent molecular weights of standards, indicated in kDa. **B.** Time-dependent changes in the intensity of the Trp-fluorescence maxima of the samples were measured, immediately after dilution into the cuvette containing McIlVaine buffer at indicated pH, and for 30 min. The ratio of the fluorescence intensity at‘t’ by the fluorescence intensity at t = 0 (just after addition of the sample into the cuvette) was plotted against time for comparison purpose. Reassembly is monitored for pH 7.0 (□), 6.7 (◊); 6.2 (▴) and 5.9 (Δ). **C.** Half-times of the reassembly reactions at indicated pH, calculated from the increase of the Trp-fluorescence intensity were plotted against pH.

CtxB_5_ was treated for 15 min at pH 1.0 and subsequently diluted to a final concentration of 8.6 µM in McIlVaine buffers at pH from 5.0 to 8.0. After incubation at different pH for 30 min, the samples were mixed with 0.1 M Tris at pH 8.0 and immediately cross-linked with glutaraldehyde, prior addition of the sample buffer (Fig. 4A).

Addition of the Tris buffer just prior addition of the glutaraldehyde, brought the pH of the reassembled samples to neutral values (6.7 to 8.0). The small variation of the pH after addition of the Tris buffer depended on the initial pH of the samples. But the neutral pH reached after addition of the Tris buffer enabled us to rule out the possibility of a lower cross-linking efficiency at acidic pH. Because of the addition of the Tris buffer, the cross-linking step happened under similar pH conditions for all the reassembled samples.

Immediately after neutralization at pH 7.0, only cross-linked CtxB monomer could be detected on the gel (Fig. 4A, 0′). In contrast, 30′ later, cross-linked CtxB pentamer, tetramer, trimer and dimer were detected in addition to the cross-linked monomer, indicating that reassembly had taken place (Fig. 4A, 30′). As the pH was lowered to 6.0, the amount of cross-linked CtxB pentamer, tetramer, trimer and dimer decreased significantly. At pH below 6.0, little cross-linked trimer was detected and it was mainly cross-linked CtxB monomer and dimer that were observed on the gel. The cross-linked dimer remains clearly detected even when the reassembly took place at pH 5.0. After 30 min reassembly, aggregated cross-linked CtxB species were also seen on the gel as previously observed, making the quantification of the CtxB intermediates unreliable [19].

The inhibition, by the low pH, of the formation of CxtB oligomeric intermediates was also observed using Trp-88 fluorescence measurements. CtxB_5_ was treated for 15 min at pH 1.0 and subsequently diluted to a final concentration of 8.6 µM, into a cuvette containing McIlVaine buffers at pH from 5.0 to 8.0. Measurements of the intensity of the Trp-88 fluorescence maxima were started immediately. The data showed an increase of the intensity of the Trp-88 fluorescence maxima with time and rising with the increase of the pH (Fig. 4B). The kinetics of this increase decreased in a sigmoid fashion with an inflection point centered at around pH 6 (Fig. 4C). So, besides quenching the Trp-88 fluorescence within the CtxB monomer (fluorescence at time 0, Fig. 3B), the protonation of histidine residue(s) also quenched the time-dependent increase of the Trp-88 fluorescence maxima during reassembly with a similar mid-point. As observed by cross-linking and SDS-PAGE analysis, the association of CtxB subunits was inhibited by the low pH (Fig. 4A). It was then concluded that the pH- and time-dependent increases of the Trp-88 fluorescence maxima was monitoring CtxB association, as shown previously for reassembly at pH 7.0 [19].The shape of the increase of the intensity of the Trp-fluorescence maxima measured for a reassembly at pH 5.9, suggested that complex kinetics took place during the reassembly. At least, two kinetics could easily be observed at this pH. The half-time was estimated for the slow reaction. The fast reaction, which also took place for a reassembly at pH 5.0 with no additional slow step, most likely involved folding steps within the CtxB monomer (unpublished results). The detailed study of these complex kinetics is currently under investigation.

So the inhibition of the CtxB subunit association was due to the protonation of histidine residues within the CtxB monomer, consequently altering its capacity to the associate. Thus, the scenario 4 was ruled.

In the scenario 1, the fold of the protonated CtxB monomer is globally disturbed and leads to misfolding of the molecule (Figs. 2A). In the scenario 2, the fold of the protonated CtxB monomer is only locally disturbed leading to the inhibition of CtxB subunit association (Fig. 2C). In both cases the protonated CtxB monomer is association-incompetent. In the scenario 3 (Fig. 2D), the fold of the protonated CtxB monomer is also disturbed but that only partially affect its capacity to associate and it can form one interface out of the two present per subunit.

In the scenari 1 and 2, the collision rate is pH-dependent as the pH determined the ratio of deprotonated to protonated CtxB monomers, but it is concentration-independent. In contrast, in the third scenario, the protonated CtxB monomer is only association-deficient and therefore the collision rate is not only sensitive to the pH (ratio of deprotonated to protonated CtxB monomers) but also to the toxin concentration. Thus, looking at the effect of the toxin concentration on the yield of reassembly for different pH conditions, would allow discriminating the scenari 1&2 from the scenario 3.

### Effect of the toxin concentration on the inhibition of the toxin reassembly by the low pH

**Figure 5:**
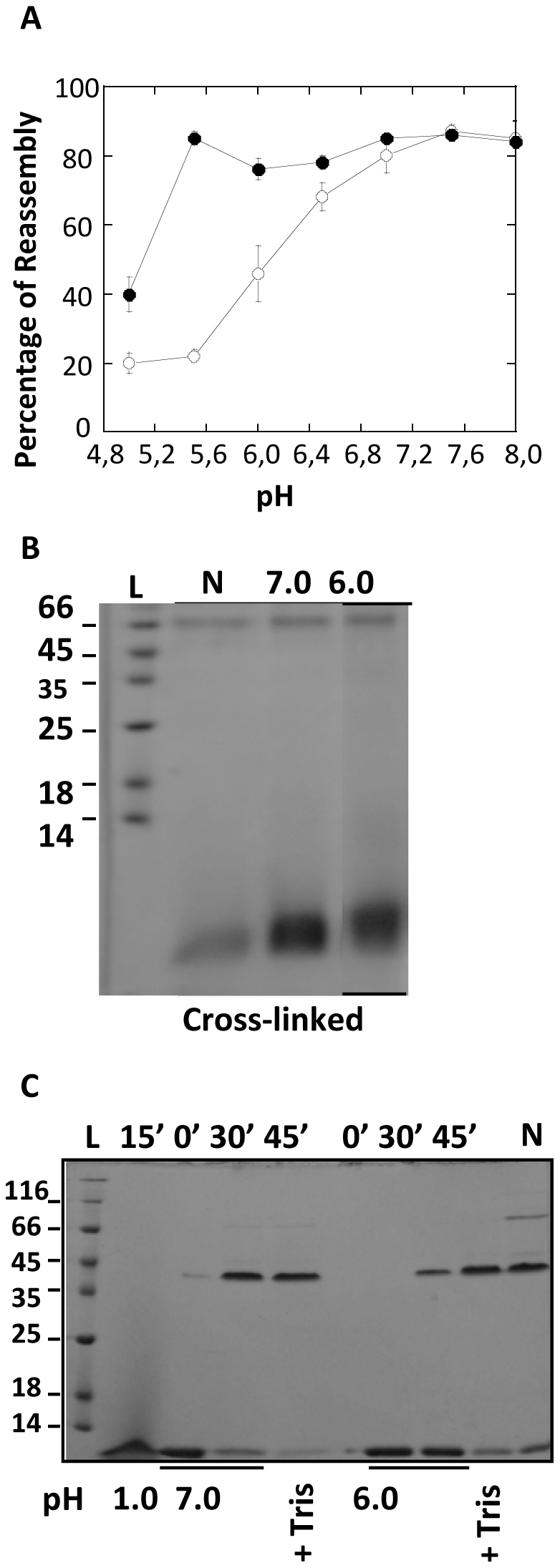
Effect of the toxin concentration on the pH-dependence of the CtxB reassembly. **A.** Yields of reassembly determined by GM1-Elisa after 30 min incubation at 8.6 µM (○) and at 35 µM (•) at indicated pH. The data for a reassembly at 8.6 µM are as in figure 1B. Each sample was assayed in triplicate and the results of three independent experiments were shown as a mean ± S.D. **B.** CtxB_5_ was treated for 15 min at pH 1.0 and subsequently diluted to a final toxin concentration of 35 µM in McIlVaine buffer at pH 7.0 or at pH 6.0 for 30 min at 23°C. The presence of CtxB pentamer and of CtxB intermediates was determined by cross-linking combined to SDS-PAGE (15%). The position of the native cross-linked pentamer (P_CL_) was identified by loading a native CtxB_5_ sample after cross-linking. M_CL_ stands for cross-linked CtxB monomer. **C.** The presence of pentamers reassembled at pH 7.0 and at pH 6.0, after a pre-treatment at pH 1.0 for 15 min, and capable of resisting SDS-treatment was monitored by SDS-PAGE (12%). The incubation time and the pH are indicated at the top and at the bottom of the gel, respectively. After 30 min incubation at pH 7.0 or at pH 6.0, 0.1 M Tris buffer at pH 8.0 (+ Tris) was added to the samples for 15 more min (45′). Native CtxB_5_ was also loaded on the gel as a marker of the pentamer position. In the gels, 1 µg of sample was loaded on each well and the gels were silver stained. L stands for the apparent molecular weight of standards, indicated in kDa.

The effect of the toxin concentration on the toxin reassembly was tested over the pH range 5.0 to 8.0. The yields of toxin reassembly monitored by GM1-Elisa at 35 µM were plotted against pH similarly to what had been performed at 8.6 µM (Fig. 5A). At 35 µM, the mid-point pH was significantly shifted to a lower pH of about 5.2 against a mid-point pH of about 6.0 at 8.6 µM. Thus, the percentage of reassembled CtxB species capable of recognizing GM1 was significantly increased with the raise of concentration. For instance, at pH 6.0, about 75% of the CtxB had reassembled into species capable of binding to GM1 against only 45% at 8.6 µM. Hence the pH-dependence of the reassembly was concentration-dependent and it was the scenario 3 that was taking place. The scenari 1 and 2 were ruled out.

### CtxB pentameric conformations

At pH 6.0 and 7.0 at 35 µM of toxin concentration, the percentage of CtxB molecules capable of recognizing GM1 was similar (Fig. 5A). We had shown previously that oligomeric CtxB intermediates, as the native pentamer, were able to recognize GM1 [19]. The CtxB species reassembled at pH 6.0 and 7.0 at 35 µM, were cross-linked and analyzed by SDS-PAGE, 30 min after reassembling to examine which of the CtxB oligomeric intermediates bound to GM1 (Fig. 5B). The CtxB intermediates were barely detectable at both pH and it was mainly cross-linked pentamer that was observed on the gel after 30 min of reassembling. Therefore, there was a striking difference in the population of CtxB intermediates reassembled at 8.6 µM and at 35 µM (Fig. 4A and 5B, respectively). For instance, at pH 6.0 little CtxB intermediate beside CtxB dimer was observed for a reassembly at 8.6 µM against a majority of cross-linked pentamer for a reassembly at 35 µM. This indicated that increasing the toxin concentration promoted the reassembly by allowing the formation of CtxB oligomeric species, step particularly inhibited at low toxin concentration. It also showed that CtxB pentamers formed at pH 6.0 and 7.0 at high toxin concentration.

The SDS-resistance of the pentamers reassembled at pH 6.0 and 7.0 at 35 µM was tested using SDS-PAGE analyses (Fig. 5C). Only little of the CtxB pentamer reassembled at pH 6.0 was able to resist the SDS treatment whereas most of the pentamer reassembled at pH 7.0 was SDS-resistant. Even after a 2H00 incubation in renaturating conditions, there was still a smaller amount of SDS-resistant pentamer formed at pH 6.0 than at pH 7.0 (not shown). This indicated that only the pentamer formed at pH 7.0 had the native conformation, capable of resisting SDS-dissociation.

When after reassembly for 30 min, 0.1 M Tris at pH 8.0 was added to the pentamers reassembled at pH 6.0 and at pH 7.0, and the samples were further incubated for 15 min, the amount of SDS-resistant pentamer formed at pH 6.0 increased to a level closed to the one observed for reassembly at pH 7.0 (with or without addition of the tris) (Fig. 5B). Similar treatment had a smaller effect on the amount of pentamer SDS-resistant reassembled at pH 7.0. It showed that by increasing the pH to neutral, the pentamer reassembled at pH 6.0 could acquire the native pentameric conformation and resist the SDS-dissociation.

This result confirmed the validity of the scenario 3 which assumed the existence of two different pentameric conformations and showed that the transition from one to the other was pH-dependent (Fig. 2D).

### Brownian dynamic simulation

In summary, three different pH-dependent steps have been identified during the reassembly of CtxB into pentamers. The first step is the formation of a CtxB monomer fully assembly competent, the second step is the association of CtxB monomers and the last one is the formation of the native CtxB pentamer. The results sustained the role of the histidine residues in the pH-dependent steps. The involvement the four histidine residues in CtxB_5_, His 13, His 18, His 57 and His 94 in the reassembly of CtxB was further investigated using the Simulation Diffusional Association (SDA) program. SDA computes association rates by simulating the diffusion association between two proteins based on Brownian dynamics (see material and methods and [26], [31], [32]. The formation of an encounter complex (association between two proteins) is defined by the establishment of any two independent native contacts in which atoms approach closer than a threshold center-to-center distance. The native contacts are the atoms closer than 4.5 Å in the native interface.

At pH 7.0, the association rate computed for two independent native contacts at an intermolecular distance of 4 Å was 3.8×10^6^ M^−1^s^−1^. This was in good agreement with previously reported diffusion-controlled protein association rates which are on the order of 10^6^ M^−1^ s^−1^ [26]. However it was a much larger value than the association rate of 3×10^3^ M^−1^ s^−1^ measured experimentally [19]. Deviation of computed association rates from experimental association rates indicates the involvement of non-diffusional rate-limiting steps during the association process, which are not accounted for in SDA [31]. The discrepancy between the experimental and the simulated association rates corroborated the implication of the isomerization of the Pro 93, a non-diffusional step, in the CtxB reassembly [19].

Using SDA, the rates of association were computed at pH ranging from 3 to 10 (not shown). At pH 3 and 4 the rate of association for two independent native contacts between atoms at an intermolecular distance of 4 Å was zero, indicating that association was not possible at these low pH. This was consistent with the experimental results that showed association was inhibited at low pH.

**Figure 6:**
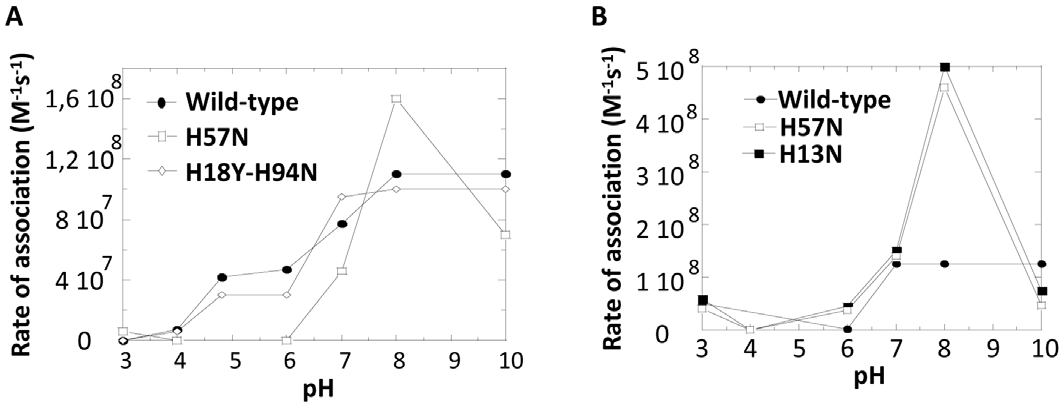
pH-dependence of the association rates determined by SDA (simulation of diffusional Association). Rates of association were computed at different pH for the wild-type toxin and for the histidine mutants by Brownian Dynamics simulation. **A. Rates of association of CtxB subunits.** For the sake of clarity only mutants H57N and H18Y-H94N are indicated on the figure. H13N and H13N-H57N gave similar results than H57N and H18Y and H94N gave similar results than H18Y-H94N. **B. Rates of association of LTB subunits.** The rates of association of wild-type and the two histidine mutants H13N and H57N are shown. The rates of association are calculated for two native independent contacts at an intermolecular distance of 6 Å.

In order to look at the effect of the pH on the wild-type association, the rates of association for two independent native contacts between atoms located at an intermolecular distance of 6 Å were plotted against the pH (Fig. 6A). This distance is about 2 Å longer than the contact distance between the atoms in the fully bound complex. At an intermolecular distance of 6 Å, the association rate for the wild-type at pH 7.0 was 7.7×10^7^ M^−1^ s^−1^, twenty times higher than the association rate computed for atoms at an intermolecular distance of 4 Å. This simply reflected the higher probability of encounters when atoms located at a larger distance are considered, consequently giving rise to higher rates of association.

As seen in figure 6, for the wild-type toxin, the association rates increased with increasing pH in a sigmoid manner with two mid-points, one acidic at around pH 4.5 and the second one neutral at about pH 7.0. Thus, the simulation reproduced the experimental results as it showed that association was pH-dependent. But in contrast to what was obtained experimentally, two mid-points were observed. The two mid-point values are different from the classical histidine pKa which is around pH 6.0.

Using PDB Viewer, mutations of histidine residues were introduced in the coordinates of the structure of the native pentamer [28]. The effect of the mutation on the association rates of two mutated CtxB monomers was tested using SDA simulations, following the same protocol than for the wild-type toxin. The native contacts involved the same atoms in the wild-type and in all the mutant interfaces showing that their rates of association were comparable (not shown).

First, the histidine residues 13 (H13N), 57 (H57N) and the two together (H13N-H57N) were replaced by asparagines and the rates of association were computed for different pH. The replacement of either H13 or H57 or of the two residues together, modified similarly and significantly the pH-dependence of the rates of association (Fig. 6A and Table 2). The mid-point at pH 4.5 was abolished whereas the neutral mid-point was still observed, at a value similar to the wild-type one, about 7.0. This suggested the involvement of the protonation of H13 and of H57 in the mid-point at pH 4.5. It also suggested that the protonation of H18 and/or of H94, the only two remaining histidine residues in the double mutant, was involved in the neutral mid-point.

**Table 2:** Mid-point pH values determined by SDA for CtxB_5_ wild-type and histidine mutants.

	1EEI	H13N	H57N	H13N-H57N	H18Y	H94N	H18Y-H94N
MP1^a^	4.5	-	-	-	4.3	3.5	4.3
MP_2_ a	7.0	7.0	7.0	7.0	6.7	6.7	6.5

a MP stands for mid-point pH.

To confirm this hypothesis, the effect of the pH on the association rates of the double mutant H18Y-H94N in which H18 and H94 had been replaced by a tyrosine and an asparagine, respectively, was tested (Fig. 6A and Table 2). This mutant has the same histidine content and the same residues at positions 18 and 94 than the wild-type LTB_5_.

The H18Y-H94N double mutant was expected to have only the mid-point at pH 4.5 but it still showed a biphasic pH-dependent curve similar to the wild-type curve, with a mid-point at around pH 4.5 and a second mid-point slightly shifted to a more acidic value than the wild-type one, to around pH 6.5. Because this double mutant only has H13 and H57, the biphasic curve necessarily implies that one is involved in the mid-point at pH 4.5 and the other in the mid-point at pH 6.5.

For the sake of simplicity, H57 and H13 would be considered involved in the mid-points at around pH 4.5 and at around pH 7.0, respectively. But the same explanation stands if the opposite is true. The simulation results were then reconsidered.

The absence of a mid-point at around pH 4.5 for the mutant H57N was obviously due to the removal of the H57 residue. The absence of a mid-point at around pH 4.5 for the mutant H13N was suggesting that somehow the pH-dependent involvement of the His 57 in the CtxB association was related to the presence of H13. The unique mid-point of the double mutant H13N-H57N indicated the involvement of H18 and/or H94 in the neutral mid-point. This was confirmed with the mutants H18Y and H94N which had a similar curve to H18Y-H94N (Table 2).

Thus, His 13 (or 57), His 18 and His 94 were involved in the neutral mid-point whereas His 57 (or His 13) was involved in the mid-point at 4.5.

Thus the BD simulations supported the role of the protonation of His 13 and of His 94 on the CtxB subunit association already suggested experimentally. They also supported the role of the protonation of His 18 and of His 57 in the subunit association, result not anticipated as these two histidines are present in LTB_5_ and are not involved in the LTB_5_ assembly [18].

The rates of association of the wild-type LTB_5_ and of two LTB_5_ mutants, H13N and H57N, were computed following the same protocol as for CtxB_5_. At pH 7.0, the rate of association of the wild-type LTB_5_ was 1.3×10^8^ M^−1^ s^−1^, a value just slightly higher than that of the CtxB_5_. The effect of the pH on these rates were tested (Fig. 6B). In contrast to CtxB_5_, the association of LTB subunit had a monophasic pH-dependence with a single mid-point at around pH 6.5 (Fig. 6B). The two LTB_5_ histidine mutants, H13N and H57N, also had a monophasic pH-dependence with mid-points slightly shifted to around pH 7. Thus, the mutation of these two histidine residues merely affected the pH-dependency of the LTB association. The two histidine mutants had a rate of association particularly increased at pH 8 for reasons which remained to be determined. This was also observed for the equivalent mutants in CtxB_5_ (Fig. 6A).

## Discussion

To investigate the role of charged residues in the slow association of CtxB monomers, the effect of the pH on the toxin reassembly was investigated.

The reassembly of CtxB into pentamers was inhibited at pH below 7.0 with an inflection point at around pH 6.0. Three different steps of the toxin reassembly were identified and found pH-dependent: (i) the CtxB monomer folding into an assembly-competent shape, (ii) the association of partially folded CtxB monomers and finally (iii) the formation of a native pentamer. Thus, a detailed mechanism of the CtxB assembly is provided and described in the model shown on Fig. 2D.

In addition, three different CtxB intermediates were identified: Two CtxB monomers and one non native pentamer. A CtxB protonated monomer, generated after acidification at pH 1.0 and subsequent incubation at pH 5.0, was found to have non native secondary and some rearrangement of its tertiary structures probably localized in the environment of the disulfide bridge linking Cys 9 and Cys 86. The conformation of this intermediate probably maintained some degree of native structure, as the proximity of histidine residues to the Trp-88 was retained and as the environment of the disulfide bridge was still constricted. The protonated CtxB monomer was association-deficient. A CtxB deprotonated monomer, generated after acidification at pH 1.0 and subsequent neutralization, was also isolated with secondary and tertiary structures both non native, as monitored by changes in its far-UV CD spectrum and in its Trp-fluorescence, respectively. The CtxB monomer formed at pH 7.0 was association-competent and recovered native secondary structures almost completely concomitantly to its assembly.

The two CtxB monomers had distinct structural elements as illustrated by their different far-UV spectra. Since it was not possible to estimate the content of secondary structures accurately, due to too high noise below 210 nm, the differences between the two monomeric conformations could not be, at this stage, evaluated more precisely.

A pentameric intermediate, which adopted a non native conformation, composed of four deprotonated and one protonated CtxB subunits, was also detected by SDS-PAGE. In contrast to the native pentamer, it was dissociated by SDS-treatment, suggesting an overall conformation different from the native one, more sensitive to dissociation, consistently with a pentamer lacking one interface, suggested by the model (Fig. 2D).

A non-native pentamer had already been reported for the B subunits of various AB_5_ toxins [12], [13], [18]. The non-native pentameric conformation was adopted after treatment of the native pentamer at pH 5.0 and did not result from disassembly/reassembly reactions. The non-native pentamer was SDS unstable and had an intrinsic fluorescence half of that of the native pentamer. In our study, at pH 6.0 and at 35 µM, about 70% of the toxin had reassembled into a pentamer SDS-unstable (Fig. 5B). The intrinsic fluorescence of that sample was 540 a.u whereas the intrinsic fluorescence of the native pentamer was 600 a.u. Hence, it was unlikely that the two non-native pentamers had the same conformation. This seems reasonable as the protonated state of the CtxB monomer within the pentamer or after a dissociation/reassociation treatment at different pH was unlikely to be similar and to have generated similar conformations.

The three different conformations of the CtxB pentamer identified are all capable of recognizing GM1 and therefore are functionally undistinguishable. It illustrates that to a unique sequence corresponds multiple conformations functionally suitable, in good agreement with the energy landscape theory [33], [34], [35].

Apart from the AB_5_, there are several other examples of non-native oligomers which have been structurally identified. There are the well-documented P22 phage tail trimer, the pore-forming toxin aerolysin, the cpn10 heptamer and the globular head of C1q [36], [37], [38], [39]. The list is non exhaustive and the formation of such non native oligomeric intermediates might be a common phenomenon.

Now, the existence of such intermediates in the cellular biogenesis of these proteins remains largely unexplored. In the context of the toxin, a CtxB pentamer in an “open” conformation (only four subunit interfaces made) might be favorable for the toxin export across the outer membrane and could play a role in the biogenesis of the toxin. The export of the whole toxin CtxAB depends entirely on the B subunit since the A subunit is not exported alone [40]. The open pentamer will also have the advantage to keep the A and B subunits associated as the binding site with A only involved three CtxB subunits. The formation and the role of an open pentameric conformation during the toxin secretion *in vivo* is, at the moment, entirely speculative.

The presence of CtxB intermediates capable of assembling or assembled in a non native fold indicates that CtxB does not conform to classical assembly mechanisms involving folding of the chains first, followed by association of almost natively folded chains [41], [42], [43]. On the contrary, CtxB folding and association are clearly concomitant as observed by the changes of the far-UV spectra of CtxB at pH 7.0 (Fig. 3C) within the time frame of the association steps (Fig. 4).

Altogether the data showed that CtxB assembled through a fly-casting mechanism, which involved association of partially folded chains. This mechanism was initially described using hypothetical kinetics of binding a single repressor molecule to a DNA site and later using association of dimeric proteins [44], [45]. Only recently experimental evidences were provided to support this mechanism in the protein assembly of the cpn10 heptamer [46]. To date, apart from the cpn10 heptamer, and now the CtxB pentamer there is no other experimental example of a protein assembling through the fly-casting mechanism. Yet as mentioned above, several non native oligomers have been identified using unfolding conditions, supporting the view that more protein oligomers are concerned by that non classical assembly mechanism.

Another mechanism, referred to as the conformational gating, describes protein association as the result of a local conformational change (folding step) providing the chain its capacity to associate [26]. It fits well with the fly-casting model as it only implies a local folding and not folding of the whole molecule to promote subunit association.

Our experimental and simulation evidences point to the role of the protonation of the four histidine residue(s) in the assembly mechanism and in particular in the regulation of the association-competency of the toxin monomer. First, the assembly, monitored by different methods is inhibited by the low pH, with a mid-point value around 6.0 and no involvement of the N-terminal protonation, supporting the role of the protonation of histidine residues instead. Second, the quenching of the Trp-88 fluorescence at low pH within the CtxB monomer is consistent with the protonation of His 94 and/or His 13, proposed by De Wolf for the CtxB pentamer at pH 5.0 [12]. Lastly, the BD simulations also indicate the role of the four histidine protonation in the toxin subunit association. BD simulations using the SDA program have been previously validated through experimental tests on several other protein systems [26], [31], [32]. The BD simulations have also indicated the role of His 18 and His 57 in the toxin association.

The role of His 18 and His 94 could be expected as these two histidines are absent in LTB_5_, whose assembly is independent of histidine protonation [18]. The independency of LTB association from His 13 and His 57 was confirmed by BD simulations. So the involvement of His 13 and His 57 exclusively in the CtxB_5_ assembly came as a surprise. It is tempting to speculate that His 13 and His 57 are recruited by the presence and/or by the protonation of His 18 and of His 94 to act together to regulate the CtxB assembly.

So we have now identified in CtxB, amino acids which are involved in some association steps, unshared with LTB. As hypothesized in the introduction, the presence of such steps can explain why the cis-trans isomerization of Pro 93 is only detectable during the CtxB assembly and not during the LTB assembly [19]. Again, it is possible that the presence of the histidines recruits the Pro-93 for regulating the CtxB assembly. The five amino acids identified are spread along the sequence and may be cross talking to regulate the CtxB assembly. These features are reminiscent of a network action.

CtxB_5_ and LTB_5_ have 86 amino acids identical out of 103. Yet, it is not enough to drive the two toxins onto the same assembly pathway. In other words, the 86 amino acids have a negligible role in the toxin assembly and it is the remaining 17 or some of them which are responsible.

There are other examples of protein for which the role of few amino acids in their folding and assembly routes have been observed. Namely, the two homologous heptameric co-chaperonin protein 10 (cpn10), from human (hmcpn10) and from the hyperthermostable bacterium Aquifex aeolicus (Aacpn10) which shares high sequence identities, have similar atomic structures yet have also been shown to follow two different refolding and reassembly mechanisms [46]. And the murine and human transthyretin tetramers which share 80% sequence identities, have similar atomic structures, yet followed different disassembly and reassembly processes [47].

It is possible that only a network of few amino acids carries out the task of assembly, whereas the rest of the amino acids are involved otherwise. This is a novel point of view as classically all the amino acids of a protein are considered when tackling the problem of protein folding or protein assembly and not only a subset. It is compatible with the robustness of protein structures to mutation described by the notion of minimal frustration [48].
